# Extraskeletal osteochondroma within the iliopsoas muscle: case report

**DOI:** 10.1051/sicotj/2017043

**Published:** 2017-09-14

**Authors:** Svetoslav Slavchev, Georgi P. Georgiev

**Affiliations:** 1 Department of Orthopedics and Traumatology, University Hospital of Orthopedics “Prof. B. Boychev”, Medical University Sofia 1 Sveti Georgi Sofiiski St. 1431 Sofia Bulgaria; 2 Department of Orthopedics and Traumatology, University Hospital Queen Giovanna-ISUL, Medical University Sofia ul. Bialo More 8 BG 1527 Sofia Bulgaria

**Keywords:** Extraskeletal osteochondroma, Iliopsoas muscle, Surgery

## Abstract

Osteochondromas, occurring usually in the metaphyses of long bones, are among the most frequent benign musculoskeletal neoplasms and both their sporadic and hereditary variants have been studied extensively. Extraskeletal osteochondromas, however, are much less common. They have been shown to arise near joints or synovial spaces of feet, hands, or bursae. Herein, we present a very rare case of an extraskeletal osteochondroma within the iliopsoas muscle.

## Introduction

Osteochondromas of long bones are among the most frequent benign musculoskeletal neoplasms and both their sporadic and hereditary variants have been studied extensively. Their extraskeletal counterpart, however, is much less common and usually arises at sites which are in proximity to synovial tissue. The most frequent locations are within or near the knee, smaller joints of the hands, feet, tendon sheaths, bursae, and sometimes in intramuscular planes [1–3]. To the best of our knowledge, there is so far only one report of an extraskeletal osteochondroma within a single muscle belly [4].

## Case report

A 42-year-old woman with type 2 diabetes and ischemic coronary disease was referred to our institution with pain in the right inguinal region with a duration of two months, swelling of the lower leg, inability to fully extend the hip joint for one month, and a palpable mass that had been noticed two weeks prior to the referral. She had had selective coronary arteriography six months earlier with ultrasound-guided access through the right femoral artery. Sonography at that time failed to detect any abnormality. No peri- or postinterventional complications had been recorded.

**Figure 1.**
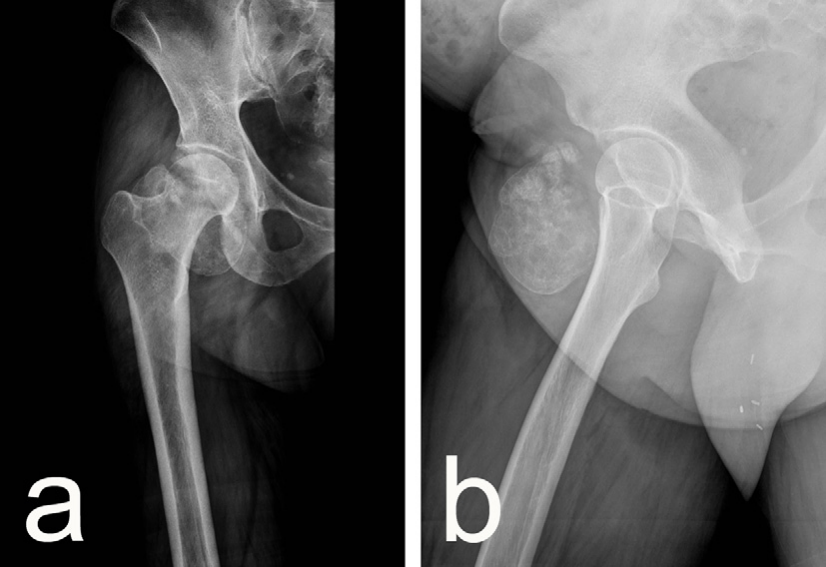
(a, b) Preoperative radiographs showed a well-circumscribed ovoid radiopaque mass.

**Figure 2.**
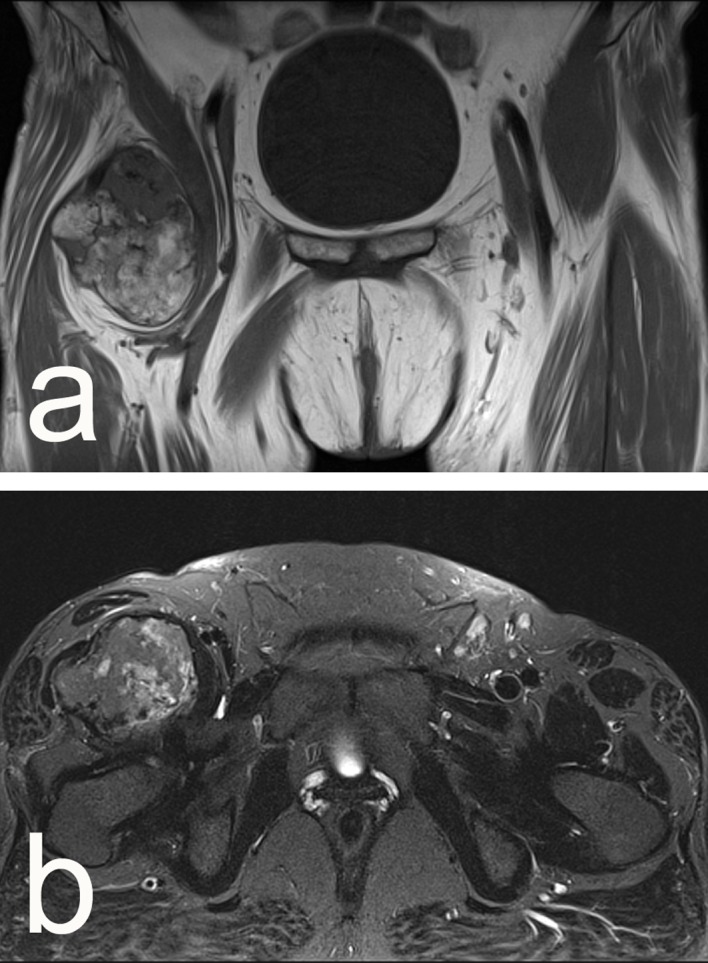
(a, b) Preoperative MRT showed a well-circumscribed ovoid radiopaque mass confined to the iliopsoas muscle belly.

**Figure 3.**
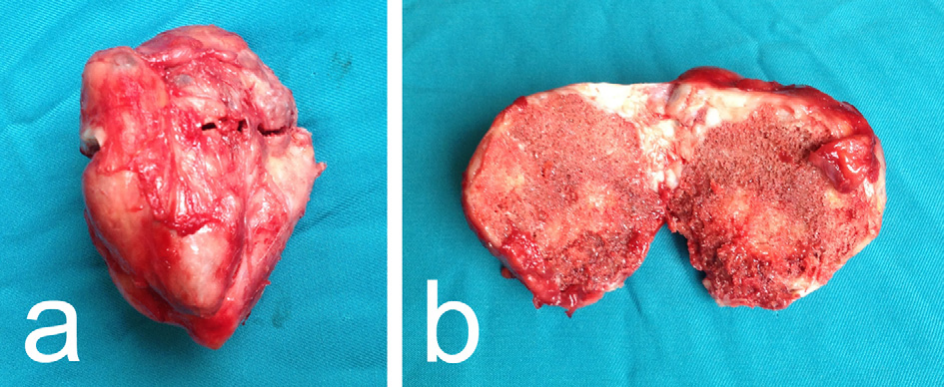
(a, b) The photograph shows the mass consisting of cancellous bone with a thin cartilage-like covering.

**Figure 4.**
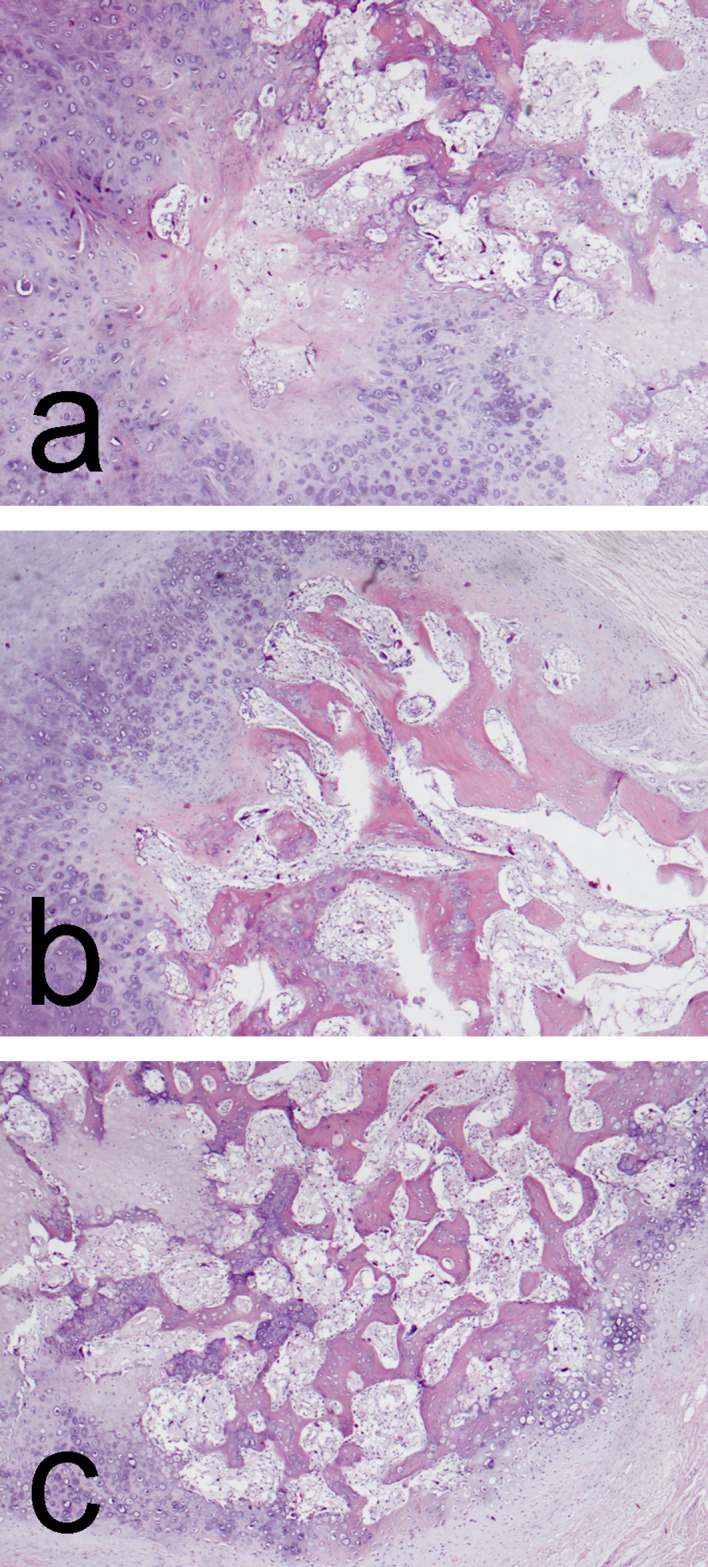
(a–c) Microscopic appearance from different parts of the material revealed a well presented cap of hyaline cartilage gradually changing into trabecular bone (hematoxylin and eosin, ×40).

Clinical examination revealed a tender, dense, hardly movable mass in the left inguinal region measuring about 10 cm by 10 cm. Pain was exacerbated by hip extension and relieved by flexion. Ipsilateral calf circumference was 1 cm greater than the contralateral calf. Plain radiography showed a well-circumscribed ovoid radiopaque mass with a structure resembling that of cancellous bone with a thin cortical shell (Figures 1a and 1b). Those findings were confirmed by magnetic resonance tomography (MRT). The MRT showed that the mass was confined to the iliopsoas muscle belly distal to the inguinal ligament with non-infiltrative growth and no perifocal edema in the surrounding muscle and it was displacing the femoral neurovascular bundle (Figures 2a and 2b). Biopsy was bypassed because of the markedly benign imaging characteristics and the proximity of the neurovascular bundle where a scarred open biopsy tract would create unnecessary difficulty in the subsequent excision. Surgery was performed through a longitudinal incision in the lateral part of the femoral triangle; the femoral nerve, artery, and vein were mobilized and retracted medially and the mass was removed from within the belly of the iliopsoas muscle by sharp and blunt dissection. The wound was closed in layers over a drain in the usual manner. The specimen had bone density and it seemed to be covered by a thin fibro-cartilaginous layer. When sectioned, it had cancellous bone structure and a thin cartilage-like covering thicker only in the proximal pole (Figures 3a and 3b). Microscopy showed a typical structure of osteochondroma with thin mature cartilage at the periphery and cancellous bone with bone marrow in the intertrabecular spaces (Figures 4a–4c). Perioperatively, standard deep vein thrombosis prophylaxis was administered and the postoperative course was uneventful apart from painless swelling of the limb that required no other treatment and resolved over the course of three weeks. There were no complications or local recurrence on 1.5 years follow-up.

## Discussion

Soft tissue osteochondromas (STO) are defined as a variant of soft tissue chondromas representing benign soft tissue tumors occurring in extra-osseous and extra-synovial locations, predominantly composed of adult type hyaline cartilage, devoid of other differentiated elements, except osseous, fibrous, and/or myxoid stroma [5].

Their etiology is unknown, trauma being inconsistently reported. Their histogenesis is still in debate. Pluripotent cells of synovial or fibroblastic origin, as well as synovial metaplasia, have been suspected to give rise to this neoplasm. In some cases, chromosomal aberrations common to other benign connective tissue tumors have been reported while in others no chromosomal abnormalities have been detected [6, 7].

The STO usually appear after the fourth decade of life in the hands or feet (82–84%) and less frequently near the knee or hip, in the thigh, buttock, skin, or other parts of the body [1, 7, 8]. They grow slowly and usually only produce symptoms when their size becomes large enough to compress nearby structures.

After an extensive review of the PubMed database, we identified one similar case published in the literature. El Samman et al. (2010) presents a case of a 44-year-old patient with complaints of increasing pain in the right groin due to extraskeletal osteochondroma treated successfully by excision [4].

The diagnosis is usually based on radiography and MRT. The tumor presents as a well-defined soft tissue radiodensity possibly with a cancellous bone structure. The MRT reveals the thickness of its cartilaginous component, hence the risk of transformation into a chondrosarcoma, its relation to critical anatomic structures and confirms its benign nature.

Histologically, the tumor consists of an outer layer of mature hyaline cartilage, areas of endochondral ossification, and different amounts of mature bone; few authors mention fatty marrow in the intertrabecular spaces [6]. Although not strictly compliant with the definition of this tumor, bone marrow colonization of the cancellous bone might represent the end stage of the natural course of the disease that is observed only rarely because of the size and duration needed for it to occur.

Apart from other benign soft tissue radiodense tumors that need the same type of marginal excision, the main differential diagnoses include chondrosarcoma, synovial sarcoma and extraskeletal osteosarcoma that require much more aggressive surgical and non-surgical treatment, but also non-tumorous conditions like myositis ossificans and tumoral calcinosis where surgery might produce more harm than good.

## Conflict of interest

SS and GG certify that they have no financial conflict of interest.
